# Sufficient iodine status among Norwegian toddlers 18 months of age – cross-sectional data from the Little in Norway study

**DOI:** 10.29219/fnr.v62.1443

**Published:** 2018-10-25

**Authors:** Inger Aakre, Maria Wik Markhus, Marian Kjellevold, Vibeke Moe, Lars Smith, Lisbeth Dahl

**Affiliations:** 1 Food Security and Nutrition, Institute of Marine Research, Bergen, Norway; 2 Department of Psychology, University of Oslo, Oslo, Norway

**Keywords:** Iodine, Urinary iodine concentration, Iodine intake, Dietary iodine intake, Toddlers

## Abstract

**Background:**

Inadequate iodine intake has been identified in several population groups in the Nordic countries over the past years; however, studies of iodine status in infants and toddlers are scarce.

**Objective:**

The aim of this study is to evaluate the iodine status and dietary iodine sources among 18-month-old toddlers from Norway.

**Methods:**

Cross-sectional and country representative data from the Little in Norway study were used. All children who had given a spot urine sample at 18 months age were included (*n* = 416). Urinary iodine concentration (UIC) was determined by inductively coupled plasma mass-spectrometry. Dietary habits and supplement use were measured by a food frequency questionnaire.

**Results:**

Median (25th–75th percentiles [p25–p75]) UIC was 129 (81–190) μg/L while estimated median (p25–p75) habitual iodine intake was 109 (101–117) μg/day. None of the children were below the estimated average requirement (EAR) of 65 μg/day or above the upper intake level of 180 μg/day. There were no differences in either UIC or estimated habitual iodine intake between different geographic areas in Norway. Milk was the most important iodine source, contributing an estimated 70% to the total iodine intake, while other foods rich in iodine such as seafood and enriched baby porridge contributed about 30%.

**Conclusions:**

The iodine status among 18-month-old toddlers from different geographic areas in Norway was sufficient, indicated by a median UIC above the WHO cutoff of 100 μg/L. This was further supported by the estimated habitual iodine intake, where none of the participants were below the EAR. Milk was an important iodine source in this age group; thus children with a low intake might be at risk of insufficient iodine intake.

Infants and toddlers are particularly vulnerable to inadequate iodine nutrition, as iodine is crucial for optimal child growth and development (1, 2) through the many functions of the thyroid hormones (3, 4). Thus, iodine deficiency has been pointed out as one of the main factors that prevent children from achieving their developmental potential (5). Even though the global work towards eliminating iodine deficiency disorders has been successful (6, 7), iodine deficiency has been reemerging in Europe (8); and inadequate iodine status has been reported in several European countries during recent years (9–12).

Iodine is present in relatively few food groups, and iodized salt is the most important source globally (13). In Norway, the permitted iodine level of 5 μg/g in table salt is too low to be considered a significant iodine source in the population (9). However, animal feed is enriched with iodine in Norway; therefore milk, dairy products, and eggs have significant levels of iodine. Marine fish, especially lean fish such as cod, haddock, and saithe, and fish products also have high levels of iodine (14, 15). Industry-manufactured baby food is enriched with iodine; thus among toddlers this is an important iodine source as well as breast milk or formula (16). Dietary surveys among Norwegian toddlers revealed that only 35% and 4% were still breastfed at 12 (17) and 24 months of age (18), respectively. Young children in the weaning period are therefore dependent on iodine-rich complementary foods in order to reach an intake of 50–70 μg/day as recommended in the Nordic countries (19).

As the consumption of milk, yoghurt, and lean fish has been declining in Norway, recent studies have reported insufficient iodine status among pregnant and lactating women (20–23). An association between insufficient iodine intake in pregnant Norwegian women and poorer developmental status in children at 3 years of age has also been found (24). Infants and young children have therefore been identified as a vulnerable group regarding insufficient iodine intake. Recently published data among 5-year-old preschool children (*n* = 220) and 3–9-year-old children (*n* = 47) showed iodine sufficiency in these groups, with a median urinary iodine concentration (UIC) of 132 and 148 μg/L, respectively (25, 26). Studies among infants and toddlers remain scarce; however, iodine status was measured in a study of Norwegian toddlers under the age of 2 with cow’s milk protein allergy. This study found a median UIC of 159 ug/L, indicating sufficient iodine status (27). The main objective of this paper is to assess iodine status in toddlers 18 months of age participating in the Little in Norway study (LiN). To our knowledge, this is the first paper from Norway to present data on iodine status and its relation to dietary habits among healthy children less than 2 years of age.

## Subjects and methods

### Study design and subjects

This paper is based on data from the LiN project (ISRCTN registry number 66710572), a prospective population-based cohort study conducted between September 2011 and November 2014. The study was established to investigate pre- and postnatal risk factors influencing child development from pregnancy to 18 months of age. Pregnant women at nine primary health clinics across all four Norwegian health regions were recruited. The data collection included questionnaires completed by the mothers and biological samples of mother and child. In total, 1,036 pregnant women consented to participate in the LiN cohort. In this paper, cross-sectional data from toddlers 18 months of age were used, as well as background characteristics of their mothers at study enrollment. Of the 1,036 participating pregnant women, 777 children were still participating at 18 months age. Not all toddlers were able to give a urine sample at the time of data collection and some failed due to technical issues. Thus, the final sample size consisted of 416 toddlers 18 months of age, along with their mothers. Further details regarding study attrition for the participants have been described elsewhere (28).

### Urinary iodine concentration

UIC was assessed in spot urine samples from the children using Uricol collection pack (Sterisets International B.V., SteriSets GP Supplies, Newcastle Urine Collection Pack, UK). The urine was extracted from the pad with a syringe and transferred to CryoTubes (CryoTubes™ Vials, Nunc A/S, Roskilde, Denmark) for storage at −18°C pending analysis. Content of iodine in urine was determined by inductively coupled plasma mass-spectrometry at the Institute of Marine Research in Norway. Further description of the analytic method and accuracy has been published elsewhere (23).

### Estimated habitual iodine intake

The children’s habitual food intake was mapped by the mothers of the children answering questions about average intake of selected food items and dishes through an online questionnaire. There were 13 questions concerning the general diet, of which nine questions concerned iodine-containing food items, where intake of yoghurt, porridge, fish, and fish products was assessed. Frequency responses were recorded as never/rarely to seven times per week or more. There was one question assessing intake of eggs, where the frequency responses ranged from less than one egg per week to eight or more per week. There were nine questions assessing intake of fats and oils, of which questions regarding margarine and butter were relevant for iodine intake. The frequency responses ranged from never to daily. There was one question regarding breast milk intake at 18 months of age, where the frequency responses ranged from once in the last 24 h to 10 times or more. However, there were no data available from Norway regarding the amount of breast milk consumed among 18-month-old children. Nor has data regarding breast milk intake been registered in the national dietary surveys for 1- and 2-year-old children in Norway (17, 18). Therefore, children still breastfed at 18 months were excluded from the iodine intake estimations. The intake frequencies related to yoghurt, porridge, fish/fish products, eggs, and butter/margarine were converted to daily amounts using data from a national nutrition survey among children 2 years of age (18) and multiplied with the iodine concentration for each food item or dish. In all calculations, iodine content reported in the Norwegian Food Composition Table (29) was used. The questionnaire did not contain intake of milk and cheese, which are important dietary iodine sources in Norway. Mean intake of milk, white cheese/cheese spread and brown cheese/whey cheese spread among Norwegian 2-year-olds, both users and non-users of the food, was used to calculate the iodine contribution from these foods. In total, milk and cheese were estimated to contribute 79 μg/day, which was applied in the estimation of daily iodine intake among all non-breastfed children.

The frequency responses of the major iodine-contributing foods – fish, yoghurt, and porridge – were divided into low/medium consumption and high consumption using the following criteria: high consumption of fish: lean fish or fish products for dinner at least two to three times per week and fish (fatty and lean) as bread topping at least four to six times per week; high consumption of yoghurt: at least four to six times per week; high consumption of fish and yoghurt: both intake of fish and yoghurt was high; high consumption of porridge: (homemade or industry manufactured) at least four to six times per week.

### Definitions of iodine status and iodine intake

The epidemiological criteria for assessing iodine nutrition based on median UIC for children were used in this study (13). For children less than 2 years of age a median UIC <100 μg/L indicates insufficient iodine status, while a median UIC ≥100 indicates adequate iodine status. In the Nordic Nutrition Recommendations, an iodine intake of 50–70 μg/day is estimated to be sufficient for infants and children <2 years of age (19). However, as the Nordic Nutrition Recommendations does not have an average requirement for young children, the estimated average requirement (EAR) from the US Institute of Medicine of 65 μg/day was used for evaluating the habitual iodine intake from food (30). To assess excessive iodine intakes, the World Health Organization’s (WHO) upper intake level (UL) of 180 μg/day for children under 2 years was used (31).

### Background characteristics and anthropometry

The mothers answered a precoded questionnaire concerning background variables for themselves and their children. The WHO body mass index (BMI) (kg/m^2^) was used to classify underweight, normal weight, overweight, and obesity, defined by BMI < 18.5 kg/m^2^, BMI = 18.5–24.9 kg/m^2^, BMI = 25.0–29.9 kg/m^2^, and BMI ≥ 30 kg/m^2^, respectively (32). The children’s height and weight were registered at the primary health clinic by trained health personnel. The gender-specific *z*-scores height-for-age, weight-for-age, weight-for-height (WHZ), and BMI-for-age (BMIz) were calculated using the WHO macro for SPSS (33, 34). A child was categorized as undernourished if WHZ or BMIz, was <−2, and overweight if WHZ or BMIz was above 2.

### Ethics

Ethics approval for the survey was given by the Regional Committees for Medical Research Ethics (2011/560 REK Sør-Øst). Written informed consent was provided by the mothers on behalf of themselves and their children. All aspects of the study agreed with the latest version of the Helsinki Declaration.

### Statistics

Normally distributed data were presented as mean ± SD. Non-normally distributed data were presented as median and 25th–75th percentiles (p25–p75). Due to the skewed distribution, non-parametric tests were used for two-sided tests of differences between groups (Mann–Whitney U test). The UIC among children was used as dependent variable in linear regression analyses. Because of skewed distribution, UIC was log2-transformed. Background characteristics (from Table 1) and dietary variables (from Table 4) were assessed for associations in simple linear models. All variables with an association (*p* < 0.20) were selected for the preliminary multiple model, which included the following: iodine supplements during pregnancy, high consumption of fish, high consumption of fish and yoghurt, high consumption of porridge. By backwards stepwise selection conducted manually, variables with a significant association at *p* ≤ 0.05 were included in the final model. Analysis of the residuals was performed to examine the fit of the model.

## Results

Background characteristics of mothers and toddlers are presented in Table 1. The distribution of participants between different geographic regions in Norway was quite even. Mean age among the mothers was 30 years, and 82% had higher education at university level. The gender distribution among the toddlers was even, with 52% boys and 48% girls. Almost 10% of the toddlers were still breastfed, 67% had received breast milk previously, while 4.6% had never been breastfed. Only 0.7 and 5.3% were wasted and overweight, respectively, according to weight-for-length *z*-scores. In total, 60.8% of the toddlers received dietary supplements, and cod liver oil was the most commonly used supplement.

**Table 1:** Characteristics of Norwegian mothers and toddlers 18 months of age

Characteristics of mothers	(*n* = 416)	Characteristics of toddlers	(*n* = 416)
Age, years	30.3 ± 4.7	Boy	217 (52.2)
BMI, kg/m^2^^a^	23.8 ± 4.5	Girl	198 (47.6)
<18.5	15 (3.6)	Never been breastfed	19 (4.6)
18.5–24.9	233 (56.9)	Stopped breastfeeding	279 (67.1)
≥25	101 (24.3)	Still breastfed	41 (9.9)
Education level		Breastfeeding frequency per 24 h^c^	
Primary and secondary school	7 (1.7)	1 time	6 (14.6)
High school	67 (16.2)	2–3 times	25 (61.0)
<4 years of university^b^	167 (40.1)	≥4 times	10 (24.4)
≥4 years of university^b^	174 (41.8)	Weight-for-length/height, *z*-score	0.6 ± 1.0
Work situation		<−2 (wasted)	3 (0.7)
Work full-time	319 (76.7)	>2 (overweight)	22 (5.3)
Work part-time	29 (7.0)	BMI-for-age, *z-score*	0.5 ±1.01
Student	58 (13.9)	<−2 (underweight)	5 (1.2)
Unemployed	9 (2.2)	>2 (overweight)	21 (5.0)
Geographic region		Supplement use (all types) weekly	253 (60.8)
Mid-Norway	134 (32.2)	Cod liver oil	151 (36.3)
North Norway	80 (19.2)	Vitamin D drops	84 (20.2)
Western Norway	89 (21.4)	Omega-3	19 (4.6)
Eastern Norway	112 (26.9)	Multivitamin mixture	40 (9.6)
Use medication for thyroid disorder	15 (3.6)	Iron	1 (0.2)
Used iodine supplements during pregnancy	91 (21.9)	Other	14 (3.4)

Values are presented as mean ± SD and *n* (%).

a Body mass index before pregnancy.

b University or university college.

c Breastfeeding frequency among children still breastfed (*n* = 41). Missing values: 67 missing from women’s BMI; 1 missing from mother’s education; 1 missing from geographic area; 1 missing from use of medication for thyroid disorder; 93 missing from iodine supplements during pregnancy; 1 missing from tobacco use in pregnancy; 1 missing from work situation; 1 missing from gender of child; 77 missing from breastfeeding status; 21 missing from anthropometric measures of children; 90 missing from supplement use in children.

**Fig. 1:**
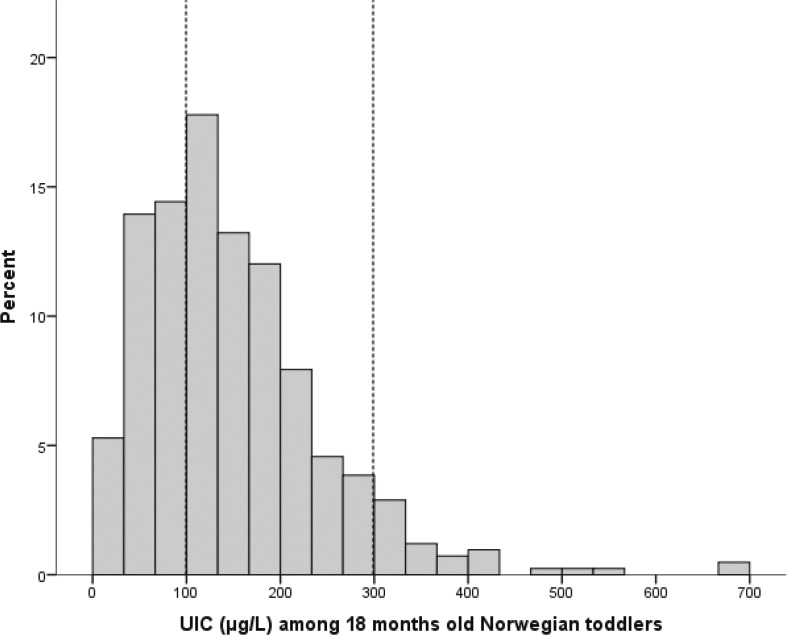
Distribution of urinary iodine concentration (UIC) among Norwegian children 18 months of age: 33% had UIC below 100 μg/L, 59% had UIC between 100 and 299 μg/L, and 7% had UIC above 300 μg/L (*n* = 416).

Table 2 presents the UIC among the toddlers in different geographical regions of Norway and across all areas. The median UIC (p25–p75) was 129 (81–190) μg/L. As indicated by Fig. 1, 34% had UIC below 100 μg/L, 59% had UIC between 100 and 200 μg/L and 7% had UIC above 300 μg/L. There were no significant or substantial differences in UIC between different geographical regions or genders. The children who had never been breastfed had higher median UIC (149 μg/L) than children who were no longer breastfed. The children who were still breastfed had a median UIC of 117 μg/L; however the differences in UIC between breastfeeding statuses were not statistically significant. Children who were attending kindergarten had similar median UIC as children who were not attending kindergarten.

**Table 2:** Urinary iodine concentration (UIC) among Norwegian toddlers 18 months of age by different geographical regions and characteristics (*n* = 416)

	UIC (μg/L)				Min	Max
	Median	p25–p75	Mean	SD		
Total (*n* = 416)	129	81–190	148	97	8	688
Gender						
Boys (*n* = 217) Girls (*n* = 198)	139 128	83–258 75–199	147 150	95 100	12 8	687 688

Geographic region						
Mid-Norway (*n* = 134)	125	69–186	138	83	8	426
North Norway (*n* = 80)	136	94–195	149	83	17	349
Western Norway (*n* = 89)	144	88–220	170	125	14	688
Eastern Norway (*n* = 112)	125	75–182	143	95	14	515

Breastfeeding status						
Never been breastfed (*n* = 19)	149	76–212	169	126	24	515
Stopped breastfeeding (*n* = 279)	130	74–201	146	91	8	539
Still breastfed (*n* = 41)	117	85–188	144	87	19	422

Kindergarten attendance						
Yes (*n* = 332)	131	81–190	148	94	8	687
No (*n* = 68)	126	73–195	148	114	16	688

There were no significant differences in UIC between gender (*p* = 0.461), geographic areas (*p* = 0.321), breastfeeding status (*p* = 0.854), or kindergarten attendance (*p* = 0, 311) tested by Kruskal–Wallis test/Mann–Whitney U test.

Intake frequencies of iodine-rich foods are shown in Table 3. Yoghurt was commonly consumed among the toddlers; however, 22% were given yoghurt ≤1 time/week. About two-thirds of the children were given porridge, either industry manufactured or homemade ≤1 time/week. About 80, 70, and 80% of the children were given fatty fish, lean fish, or fish products for dinner ≤1 time/week, respectively. Baby food with fish was not commonly consumed, with 95% in the category of less than or equal to once a week. Lean fish products such as caviar and fishcakes and fatty fish products such as mackerel or salmon were consumed as bread spread among 31 and 36% 2–3 times/week or more frequently, respectively, while the rest consumed fish as bread topping ≤1 time/week. Sixty-eight percent consumed eggs less than or equal to once a week.

**Table 3:** Frequency of intake (times/week) of iodine-rich foods among Norwegian toddlers 18 months of age (*n* = 340^a^)

Iodine-rich foods	Never/rarely	1 time per week	2–3 times per week	≥4 times per week
	*n* (%)	*n* (%)	*n* (%)	*n* (%)
Yoghurt	41 (12)	35 (10)	95 (28)	169 (50)
Porridge (industry manufactured)	193 (57)	28 (8)	37 (11)	82 (24)
Porridge (homemade)	147 (43)	85 (25)	62 (18)	46 (14)
Fatty fish, dinner	69 (20)	202 (59)	67 (20)	2 (1)
Lean fish, dinner	80 (19)	221 (53)	39 (12)	0
Fish products, dinner	55 (16)	225 (66)	55 (16)	5 (2)
Baby food with fish (industry manufactured)	296 (87)	28 (8)	15 (4)	1 (0.3)
Fatty fish, spread	171 (50)	64 (19)	64 (19)	41 (12)
Lean fish, spread	141 (42)	78 (23)	87 (26)	34 (10)
Eggs	86 (32)	96 (36)	63 (24)	22 (8)

Values given in *n* (%) within participants with dietary intake category.

a *n* = 340 for all foods except eggs where *n* = 267.

UIC according to low/medium and high consumption frequencies of iodine-rich foods is shown in Table 4. There were no substantially or statistically significant differences in UIC between the different consumption categories for any of the foods. There were quite a few children with a high consumption frequency of fish (20%). Yoghurt was more frequently consumed, and 50% had a high intake. Only 12% had a high frequency in intake of both fish and yoghurt. Industry-manufactured porridge is enriched with iodine in Norway; nevertheless, there were no difference in median UIC between the group who received homemade porridge and those who received industry-manufactured porridge.

**Table 4:** Urinary iodine concentration (UIC) among Norwegian toddlers 18 months of age with low/medium consumption frequency and high consumption frequency of iodine-rich foods (*n* = 340)

	UIC (μg/L)			
	Low/medium consumption		High consumption^a^	
	Median (p25–p75)	*n*	Median (p25–p75)	*n*
Fish	134 (83–200)	271	117 (56–200)	69
Yoghurt	123 (79–200)	171	132 (75–200)	169
Fish and yoghurt	131 (81–200)	298	117 (55–198)	42
Porridge, industry-manufactured	129 (81–193)	258	123 (55–208)	82
Porridge, homemade	129 (76–205)	294	119 (73–185)	46

Values given as median (p25–p75).

a Fish: Lean fish or fish products for dinner at least 2–3 times/week, and lean fish or fatty fish as bread topping at least 4–6 times/week. Yoghurt: at least 4–6 times/week. Fish and yoghurt: both intake of fish and yoghurt was high. Porridge (homemade or industry-manufactured): at least 4–6 times/week. Differences in UIC between consumption frequencies were tested by Mann–Whitney U test for each of the food groups. None were statistically significant.

All the dietary variables among children included in Table 4, as well as background characteristics of the mothers and children, were tested for associations in linear regression models. None of the food consumption variables or background characteristics for mothers or children had a significant association with the children’s UIC. Table 5 shows the estimated habitual iodine intake from the main dietary iodine sources (yoghurt, milk, cheese, fish/fish products, porridge, eggs, butter/margarine) among non-breastfed children 18 months of age in Norway. Estimated median (p25–p75) habitual iodine intake was 109 (101–117) μg/day for all children across geographic regions (where the estimated iodine contribution from milk and cheese is included). There was no substantial difference in estimated habitual iodine intake between the different geographic regions. None of the children were below the EAR (65 μg/day) or above the UL (180 μg/day). Estimated iodine intake from milk and cheese contributed about 72% of the total iodine mean intake, while fish contributed about 12% and other foods about 16%.

**Table 5:** Estimated habitual iodine intake among non-breastfed Norwegian toddlers 18 months of age in different geographical regions of Norway (*n* = 232^a^)

	Estimated habitual iodine intake (μg/day)					
	Median	p25–p75	Mean	SD	Min	Max
Total (*n* = 232)	109	101–117	110	13	82	157
Geographic region						
Mid-Norway (*n* = 76)	110	101–116	109	11	90	157
North Norway (*n* = 44)	105	97–120	105	12	84	149
Western Norway (*n* = 51)	107	101–118	109	13	82	138
Eastern Norway (*n* = 61)	113	102–124	114	14	88	145

a 149 missing from dietary data and 35 excluded as they were still breastfed. Iodine intake from milk and cheese have been estimated based on data from 2-year-old children (18) and were estimated to contribute 79 μg iodine/day.

## Discussion

The Norwegian toddlers in this study had adequate iodine status, as indicated by a median UIC of 129 μg/L, which is above the WHO cutoff of 100 μg/L. This finding was supported by the estimated habitual iodine intake, which was 109 μg/day. None of the children had an estimated habitual iodine intake below the EAR of 65 μg/day or above the UL of 180 μg/day. There were no substantial differences in either UIC or iodine intake between different geographic areas of Norway. These findings are in line with the local small-scale Norwegian studies among toddlers with cow’s milk protein allergy and young children presented in the introduction (25–27).

Infant formula is enriched with iodine in Norway, and the average iodine content of several products of prepared formula intended for consumption from 6 months age, using data from the food composition table, is 15 μg/100 g (29). A recent study among lactating Norwegian women found that the median iodine concentration in breast milk was 68 μg/L (7 μg/100 g) (20), about half of the iodine content found in formula. The children who were never breastfed might still receive formula at 18 months of age, which could explain why the median UIC was highest in this group. A correlation with UIC and use of infant formula has been reported by others (35, 36). Similar results to ours were also found in the mentioned study of 57 infants under the age of 2 with cow’s milk protein allergy, where the breastfed children had lower UIC than the children who received formula or were weaned (27).

Intake of milk, formula, and cheese was not recorded in this study, which is a major limitation to the dietary data, as milk is an important component in the diet of young Norwegian children (17, 18). In our study, yoghurt was the most frequently consumed iodine-rich food. Fish and fish products were not as frequently consumed and about 52% consumed fish or fish products (all types) for dinner no more than once a week and 42% as bread spread no more than once a week (data not shown). Similar results were found in a Norwegian study among preschool children (4–6 years of age); however, the consumption of fish as bread spread was higher than in our study (26). Portion sizes were not registered in this study; however data from all the Nordic countries suggest that fish consumption is generally low among preschool children, including Norway (37). This is in line with our findings, where the majority had an intake below the recommendation for 2-year-old children (38). We did not find any substantial difference in UIC between different consumption frequencies of yoghurt, fish, porridge, or eggs, which was in line with the findings from the regression model where none of the dietary intake variables were associated with UIC. This suggests that milk probably made a large contribution to the total iodine intake among the children in this study. Milk as an important contributor to young children’s iodine status in Norway has also been found by others (25). Recent studies suggest that pregnant and lactating women in Norway have mild to moderate iodine deficiency (20–23). Others have also found adequate iodine status among children, while mothers from the same population were iodine deficient (39–41). In line with our findings, this has been suggested to be caused by a relatively higher consumption of milk among children (25, 39). On the other hand, as pointed out by Trøan et al., children with a high milk consumption may be at risk of excessive iodine intakes in Norway as the iodine content of milk is relatively high (42).

The median estimated habitual iodine intake was lower than the median UIC. There are several challenges related to dietary assessment, in addition to the mentioned limitation of using extrapolated intake values for milk and cheese. Only the main dietary iodine-containing food items were included in the estimated habitual intake, and missed sources (e.g. vegetables, meat, and bread) cannot be ruled out. Portion sizes from a national survey among 2-year-old children were used. As dietary habits rapidly change in the weaning period (43), the portion sizes may not be accurate for toddlers 18 months of age. Also, most toddlers in this study were attending kindergarten (79.8%, data not shown), which may further complicate dietary assessment as the parents have less control of food consumption. Nevertheless, the estimated habitual iodine intake indicated sufficient iodine status in this age group, which was in line with the WHO epidemiological criteria for assessing iodine nutrition by median UIC. Further, the estimated habitual iodine intake allows one to assess the iodine status at an individual level, as opposed to the UIC, which provides valuable information. None of the children had an estimated iodine intake below the EAR or above the UL.

## Conclusion

Iodine is a crucial nutrient during the first 1,000 days of life (44), and children under 2 years of age have been identified as a particularly vulnerable group for inadequate iodine intake by the WHO (31). Thus, the iodine status among young children should be carefully monitored. This study showed that the iodine status among 18-month-old toddlers was sufficient. Further, milk seemed to be the major iodine-contributing food item among these children, and the intake of fish and enriched porridge was low. Children with low intake of milk could therefore potentially be at risk of insufficient iodine intake in Norway.
